# Determination of field capacity in the Chibunga and Guano rivers micro-basins

**DOI:** 10.12688/f1000research.28143.1

**Published:** 2021-03-03

**Authors:** Benito Mendoza, Manuel Fiallos, Sandra Iturralde, Patricio Santillán, Nelly Guananga, Jaime Bejar, Daniel A. Lowy, Imre Vágó, Zsolt Sándor

**Affiliations:** 1 Chimborazo National University, Riobamba, Ecuador; 2 Fujian Agriculture and Forestry University, Fuzhou, China; 3 Escuela Superior Politécnica de Chimborazo, Riobamba, Ecuador; 4 Genesis Sustainable Future Ltd., Sárospatak, Hungary; 5 Institute of Agrochemistry and Soil Science, University of Debrecen, Debrecen, Hungary; 6 Research Group of Applied Plant Glycobiology, Dama Research Center limited, Kowloon, Hong Kong

**Keywords:** Field capacity, maximum retention potential, precipitation-runoff relationship, curve number method

## Abstract

**Background: **The micro-basins of the Chibunga and Guano rivers are located within the sub-basin of the Chambo River, which starts at the thaw of the Chimborazo, crosses the cities of Guano and Riobamba, and ends in the Chambo River. These rivers are considered fluvial hydrological forces and geological limits of the aquifer, located in this sub-basin. For this reason, our investigation addressed the field capacity in the micro-basins of Chibunga and Guano rivers, to determine the maximum retention potential, i.e., the saturation of water in the soil.

**Methods: **We investigated the change of precipitation to runoff through the correlations between the characteristics of the soil and its vegetation. We applied the Curve Number (CN) method introduced by the *United States Soil Conservation Service* (USSCS); this represents an empirical model, which relates the vegetation cover to the geological and topographic conditions of the soil. Along with the geographic information system, the model allows to represent the variation of runoffs for each micro-basin, according to the different land use categories, over the time frame from 2010 to 2014.

**Results: **We found that the maximum retention potential is directly affected by CN values, representing the runoff potential. Highest values of 100 belong to the wetlands, urban area, snow, and water, as rain is converted directly into runoff, being impervious areas. The Guano river micro-basin possesses clay soil with CN of 78, the soil texture for eucalyptus forest is clay loam, and its CN value, 46, is the lowest of the data set. Knowledge of field capacity allows to properly evaluate the storage capacity of soil and water conservation.

**Conclusions: **Results of this work will be useful in the quantification of the water balance, to determine the water supply and demand.

## Introduction

The most important natural resources are soil ( Bautista *et al*., 2018; Mátyás *et al*., 2018; Singla *et al*., 2018; Sukiasyan & Kirakosyan, 2020), air ( DRC, 2020), and water ( Bersosa *et al*., 2019). Therefore, protection of natural resources and their rational use are essential to provide sustainable environmental and agricultural management practices ( Mátyás *et al*., 2020; Sándor *et al*., 2020; Shomana *et al*., 2020). The hydrological cycle is the process of water circulation in the earth, therefore, it is important to study surface and underground water, to understand the cycle in a global way and its interrelation with the environment ( Pacheco Moya *et al*., 2018).

Also, the management of a basin must be oriented to the rational use of its resources, i.e., to order and spatially distribute their use ( Sánchez-Cohen *et al*., 2015). Management is important, as a basin has environmental, social, economic, and cultural implications in the distribution of water resources ( Sánchez-Cohen *et al*., 2015). As of today, in Ecuador, most of the hydrographic basins do not have proper conservation management, causing alterations in the hydrological cycle and generating for a given area little retention of humidity, erosion, and flooding over periods of rain and water scarcity in dry periods ( Lizárraga-Mendiola *et al*., 2019).

Surface runoff is part of the hydrological cycle; it occurs as a result of excess water, which that does not filter off, and collects in the secondary channels, and finally ends up in the main channel, until it reaches the mouth of the river ( Francés & Montalvo, 2017). When this process is not adequately controlled, it becomes a critical environmental problem, as it favors water erosion of the soil, rivers overflow, increasing the sediment load, and, by dragging, it causes water contamination ( Alonso-Brito, 2016). Various study techniques have been developed, both empirically and in the field ( Maidment *et al*., 1994), which allow determination of the volume of runoff in a given area, for providing adequate water management ( Amy, 1974). These techniques enable appropriate design structures for the use and control of water resources. Surface and underground runoffs depend on the hydraulic characteristics of the subsoil, such as hydraulic conductance and porosity, which are distributed according to the geological and topographic soil conditions ( Lee *et al*., 2015).

As a result, runoff calculations can be derived from precipitation events, using correlations between the characteristics of the soil and its vegetation, and also taking into account the interaction of different components of the natural system ( Pacheco Moya *et al*., 2018). Nevertheless, the hydro-meteorological information in the hydrographic basins is limited in terms of a rainfall and river database of several years, as there is no good hydro-meteorological network in Ecuador ( Ochoa-Tocachi *et al*., 2018), and this data is needed for hydrological modeling of water balance, when calculating water supply and demand ( Zubieta *et al*., 2018).

Therefore, one can apply empirical models that provide approximate data on the hydrological reality of an area, e.g., for runoff the Curve Number (CN) method is used ( Uwizeyimana *et al*., 2019).

The goal of this work is to determine the field capacity, considering the physical characteristics of the environment and the use of the soil and vegetation cover. We aim to obtain quantitative values for CN, and to determine the maximum potential retention and the precipitation-runoff relationship. These calculations are done for the Chibunga and Guano rivers micro-basins, located in the province of Chimborazo, Ecuador. This work should be helpful in quantifying the water balance in a basin, and to determine water supply and demand in a more efficient manner.

## Methods

### Description of study area

Our study was conducted from January 7 to March 29, 2019, in the area of interest, namely, the micro-basins of the Chibunga and Guano rivers, which are part of the sub-basin of the Chambo River, in the province of Chimborazo. Economic activity, which predominates in the region is agriculture, providing living means for most families residing there. Chibunga river originates from the slopes of the Chimborazo Volcano and descends through the Arenal moorland, until it reaches agricultural areas in San Juan town, and the communities including Chimborazo, Shobol Llinllin, with the name arising from Rio Chimborazo. Next, it joins the Cajabamba river at 3,238 m above sea level, and becomes Chibunga river ( Mayorga & Carbonel, 2018). This micro-basin extends over 38 km, extends from northwest to southeast, and is the main tributary of the Chambo River at the Northern extremity of the Amazon region of Ecuador (at 680 km from the Amazonas River) ( CNRH, 2007). The Guano River micro-basin is born on the slopes of the Chimborazo Volcano and receives contributions to its channel from streams, such as Cascajal, Chuquipogio, Abras, Puluchaca, Patulú, Igualata, Asaco, and others ( Cevallos Gaibor, 2015). Its approximate area is 37,061 ha, and most of its water is used in the Guano Canton ( Mendoza, 2016) until it eventually flows into the Chambo River.

### Identification of the sampling points

Sampling points were identified by the over position of geology and elevation layer, where the most representative categories concerning land use were identified: Pa (moorland), Hu (wetlands), Bs (forest), Na (snow and water), Z (urban area), C (crops), Se (eroded soil), and Ps (grass).

Subsequently, the following criteria were considered to determine the sampling points:

- Areas with steep slopes and poor accessibility were excluded
- Areas with a high degree of water for field tests were included
- Categories of urban area, snow, and water were excluded because of their impervious surface.

### Obtaining the ***needed*** parameters for CN

The CN parameter measured in the field (in-situ) and laboratory. First, the infiltration capacity was determined by infiltrometer (model 09.04, SDEC France, France). Second, soil characterization was done for permeability, texture, porosity, and organic matter ( Tailor & Shrimali, 2016).

## *In-situ* analysis

At each sampling point, infiltration tests were performed with by means of a double ring infiltrometer (Model 09.04, SDEC France, France). Briefly, two concentric rings were set up on a portion of soil and water was poured into the outer ring. Variation of the water level in the inner ring was measured to determine infiltration of the soil ( Carreras Nampulá *et al*., 2015). Samples should be as little disturbed as possible, for not altering the results.

### Analysis in the laboratory

**Figure 1.**
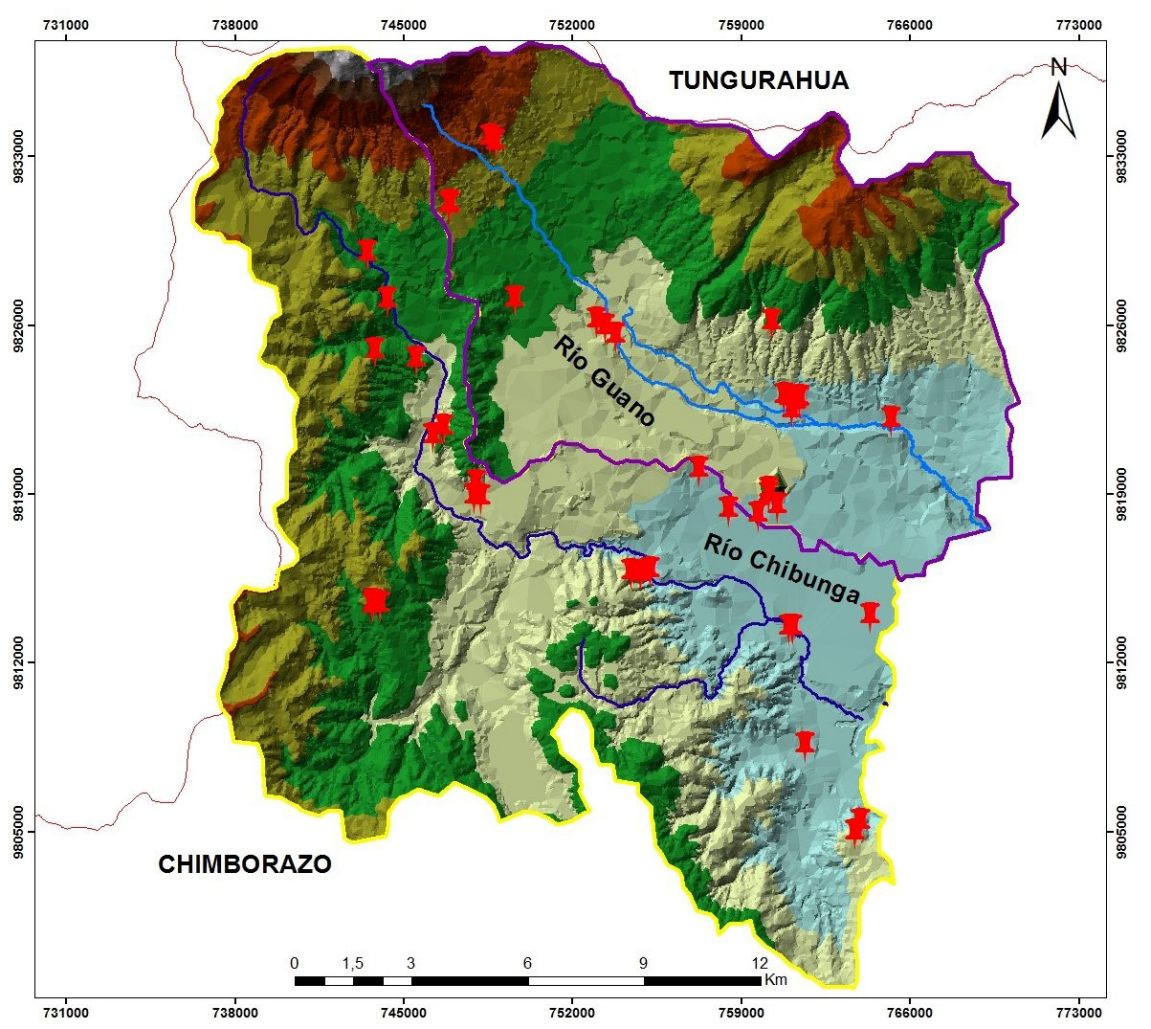
Location of the Chibunga and Guano rivers micro-basins. The red pins correspond to the Chibunga and Guano river micro-basins. Figure 1 was adapted from satellite images to identify the categories of land use and vegetation cover. These images were downloaded from the Copernicus website, using the Sentinel 2A satellite ( Immitzer *et al.*, 2016) (data is open source once a user registers for access).The images are from 2013, 2014, and 2018.

Figure 1 shows a total of 23 points corresponding to the Chibunga river micro-basin and 21 points for the Guano river micro-basin, which were used for sampling. Five soil samples were taken from each sampling point, with the excavation being every 0.40 m to a maximum depth of 2.0 m, within a circular area 0.50 m in diameter. A total of 2 kg of soil were taken by a tubular soil sampler (15”L x ¾”Dia, Accuproducts International, USA) for each point, were placed in sampling bags (125 mm × 225 mm, Geology Superstore, United Kingdom), and transported to the Environmental Services Laboratory to determine the permeability, texture, porosity, and organic matter.

Soil texture was determined according to the USDA triangle of textural classes ( Bautista Carrascosa & Oliver Talens, 2017), applying the organoleptic method. Briefly, numerical values were assigned within the range of 1–12 to define granulometry: lower numbers (1–5), represent fine granulometry, such as clay and loam clay type; mid numbers (6–9) correspond to medium granulometry, such as loams and silts soils; while high numbers (10–12), are for coarse granulometry, such as sands.

Porosity was obtained by a literature method ( Liu *et al*., 2017), which relies on the real and apparent density of the soil. Calculations were based on Equation (1), which can be applied to each soil class, identified in the study area.

$$\;P\;[\%]=\left(1-\frac{Da\left[\frac{g}{cm^{3}}\right]}{Dr\left[\frac{g}{cm^{3}}\right]}\right)\;*\;100\;\text{Eq}\;(1)$$

Where:

P = Total porosity of the soil sample, [%]

Da = Apparent density of the soil, [g/cm ^3^]

Dr = Real particle density, [g/cm ^3^]

Permeability or hydraulic conductivity ( Papierowska *et al*., 2018) was calculated using a permeameter (with internal diameter D = 6.4 cm, height L = 15.3 cm) (Cotecno Falling Head Permeameter, Santiago, Chile), in which *h* values are measured, obtained for various times elapsed from the beginning of the test. Then, the soil permeability coefficient, *k*, is calculated using Darcy's Law, according to Equation (2) and Equation (3), ( Wang *et al*., 2020):

$$\;Q=\frac{H_{3}-H_{4}}{L}\;*A\;*\;k\;(2)$$

$$\;k=\frac{Q*L}{(H_{3}-H_{4})*A}\;(3)$$

Where: Q = flow, expressed in m ^3^/s; L = length of sample (m); k = Darcy's permeability coefficient, variable as a function of the sample material, expressed in m/s; A = cross-sectional area of the sample (m ^2^); H _3_ = height, above the water level in reference tube placed at the entrance of the filter layer; H _4_ =height, above the reference level, that reaches the water in a tube placed at the out of the filter layer.

Organic matter was determined via calcination of the samples to later calculate the weight loss, which corresponds to the organic compounds present in the samples ( Papierowska *et al*., 2018). The following procedure was used: crucibles with samples were measured in triplicate. Samples were subjected to 105 °C for 2 h; then, the temperature was raised to 550 °C for 2h. At the end, samples were cooled for 2 h inside the muffle. Subsequently, samples are returned to the oven at 105 °C for 30 min, to stabilize their temperature and eliminate moisture remained in the samples, then cooled in the desiccator, and finally weighed for determining the weight loss of each sample. Soil organic matter (SOM) was determined gravimetrically and calculated according to ( Equation 4).

SOM%=[(Weightat105°C)−(Weightat550°C calcination)/(Weightat105°C) ].100(4)

### Curve number calculation (CNRH)

CN was determined according to the SCS method, a technique developed for infiltration proofs depending on the runoff generating properties. The procedure considered: the hydrological group of soil (HGS), the previous humidity condition, the use of the land and treatment within the hydrographic basin. A value is calculated, which estimates the soil condition with regard to its usefulness ( Satheeshkumar *et al*., 2017). CN is a curve number, obtained from cross-tabulation in spatial digital format, processed by soil and geomorphology maps, generating the hydrological groups (GH), as described in the *SCS Handbook of Hydrology* (NEH-4), Section-4 (USDA 1972). CN can be expressed by Equation (5).

$$\;CN=\frac{25400}{\left(254+S\right)}\;(5)$$

     where:

$$\;S=\frac{25400}{CN}-254\;(6)$$

Taking as the input values of the CN parameter, the field capacity or maximum retention potential (S) was determined as the relatively constant amount of water contained by a saturated soil, after 48 h of drainage ( Wróbel & Boczoń, 2020). Hence, there is a relationship between the soil and the coverage conditions within the micro-watersheds, via CN values. Numbers are shown on a scale from 0 to 100; the greater the number, the greater the direct runoff volume that can be expected from a storm ( Lian *et al*., 2020).

### Rainfall-effective runoff ratio

Precipitation values were obtained from the interpolation by the kriging method, by using data from 6 meteorological stations ( Table 1), over the time encompassing years 2010–2015. Precipitation values can be calculated using Equation (7) ( Naranjo, 2014)

**Table 1.** Precipitation values provided by meteorological stations between 2010 and 2015.

Id	code	Stattion name	x	y	2010	2011	2012	2013	2014	2015
1	H0786	Guamote aj Cebadas	763003	9792069	1389.55	1092.22	1433.7	1048.05	1219.11	1429.52
2	H0333	San Lorenzo en san Lorenzo	722749	9813081	1265.69	1184.1	1337.9	928.23	917.57	1852.97
3	H0787	Alao en hda. Alao	776829	9792312	995.39	1046.21	1040.52	1101.64	786.65	1693.36
4	H0788	Puela aj Chambo	780994	9832690	1551.5	1576.63	1611.57	1604.05	1589.55	2480.86
5	H0789	Guargualla aj Cebadas	766422	9792692	807.94	990.75	1013.13	1061.11	714.824	1370.87
6	H0790	Cebadas aj Guamote	762724	9791230	921.985	1020.37	861.78	787.13	762.901	1246.18

$$\;\hat{Z}(S_{0})=\sum_{i=1}^{N}\lambda_{i}\;Z(S_{i})\;(7)$$

Z (s _i_) = the value measured at location

λ _i_ = an unknown weight for the value measured at location

S _0_ = the location of the prediction

N = the number of measured values

Similarly, for runoff, the maximum retention potential was analyzed as a function of yearly precipitation. By this, one obtained the total flow, based on the vegetation cover ( Sikorska *et al*., 2018).

For the multitemporal study the following methodology was used, all methods described below being applied for each sampling site:

**Supervised Classification Criteria:** Study areas should match the number of categories that are intended to be analyzed ( TELEDET, 2017). The areas must be correctly identified to cover all classes and must be homogeneous. Also, in supervised classification uniband statistical analysis verifies that values resemble normal distribution. To meet these criteria, we used the following classic algorithms: classifier by minimum distance, by parallelepipeds, and by maximum probability ( Acosta, 2017).

## Classification methods

### Minimum Distance

With this classifier collected data is used to determine the mean of the classes selected at the study sites. This approach assigns each unidentified pixel to the class with the closest mean. For the closest mean values one can use the Euclidean distance between the pixel and the center of each class. Although simple from the computational standpoint, this algorithm has certain limitations, including the insensitivity to different degrees of variance in the spectral responses ( TELEDET, 2017).

### Maximum Likelihood (ML)

ML or maximum probability assumes that the reflectivity values in each class follow a multivariate normal probability distribution. The vector of means and the variance-covariance matrix are then used to estimate the probability by which one given pixel belongs to all classes. Finally, pixels were assigned to the class to which it belonged with the highest probability ( Tso & Mather, 2009). A Bayesian approach was used and considered was the a priori probability, whether a pixel belonged to a certain class. The greater the surface area, the greater the probability that a pixel belongs to it ( Toro *et al*., 2013).

### Contingency table

It consists of a double-entry table, which compares the real values with results obtained in the classification. The diagonal of the matrix reveals the number of real pixels and the classification that coincides by category, while the remaining pixels indicate the ones that may be confused with other categories. One represents vertically the percentage of real pixels that were confused in the truth-terrain, while horizontally one shows the percentage of all pixels ( Borràs *et al*., 2017).

### Kappa coefficient

The accuracy measures considered by us are simple to use, being based on either the main diagonal, or the rows and columns of the contingency table. In some cases, a totally random distribution of pixels in the classes can yield apparently correct results in the contingency table ( TELEDET, 2017).

## Results and discussion

Through the multi-temporal study and the application of spectral signatures, eight categories of vegetation cover were identified for each micro-basin: Pa (moorland), Hu (wetlands), Bs (forest), Na (snow and water), Z (urban area), C (crops), Se (eroded soil), and Ps (grass). The 26 sampling points were in the Chibunga river micro-basin and 23 points in the Guano river micro-basin.

Physical characteristics of the soil from each sampling point were determined, including porosity, permeability, and infiltration capacity ( Table 2). These unique values are required to determine vegetation cover and soil texture, necessary to calculate CN.

**Table 2.** Physical characteristics of the soil of the Chibunga and Guano rivers micro-basin.

Chibunga river micro-basin				Guano river micro-basin		
Class	Poro.	Perm.	Cap. Infil.	Poro.	Perm.	Cap. Infil.
Pa	0.45	2.3E-04	96.5	0.58	3.1E-04	183.2
Hu	null	null	null	null	null	null
Bs	0.58	1.2E-04	592.4	0.52	1.5E-04	60.2
Na	null	null	null	null	null	null
Z	null	null	null	null	null	null
Se	0.53	2.8E-05	120.3	0.48	2.2E-04	230.4
C	0.35	5.4E-04	118.6	0.50	3.2E-04	45.8
Ps	0.44	1.1E-04	108.7	0.53	1.6E-04	174.4

Pa (moorland), Hu (wetlands), Bs (forest), Na (snow and water), Z (urban area), C (crops), Se (eroded soil), and Ps (grass). Null values mean 0 or minute areas. Abbreviations: Poro., porosity; Perm., permeability; Cap. Infil., infiltration capacity.

Table 3 lists the values for CN and HSG (hydrological group) depending on the state of conservation and conditions present in the sampling area.

**Table 3.** Assignment of hydrological group (HSG) and curve number (CN) for the Chibunga and Guano river micro-basins.

Chibunga river micro-basin				Guano river micro-basin		
Class	Texture	HSG	CN	Texture	HSG	CN
Pa	Silty clay	D	78	Clay	D	78
Hu	Null	Null	100	Null	Null	100
Bs	Silty clay	D	79	Clay loam	D	46
Na	Null	Null	100	Null	Null	100
Z	Null	Null	100	Null	Null	100
Se	Clay loam	D	88	Silty clay loam	D	88
C	Clay	D	89	Sandy clay	D	80
Ps	Silty clay	D	79	Sandy clay	D	78

Abbreviations: Poro., porosity; Perm., permeability; Cap. Infil., infiltration capacity.

In Table 3 one finds different combinations of vegetation cover and the HSGs of the soil in the micro-basin of the Chibunga River, where each combination corresponds to a CN that represents the runoff potential, in which the highest values belong to the wetlands, urban area, snow, and water, assigning them the value of 100. In these areas rain is converted directly to runoff, as they are impervious areas. By contrast, the Guano River micro-basin revealed that the moorland class had clay soil with CN of 78, the soil texture for eucalyptus forest was silty clay, and its CN was the lowest of the data set (46).

### The maximum retention potential (S)

**Figure 2.**
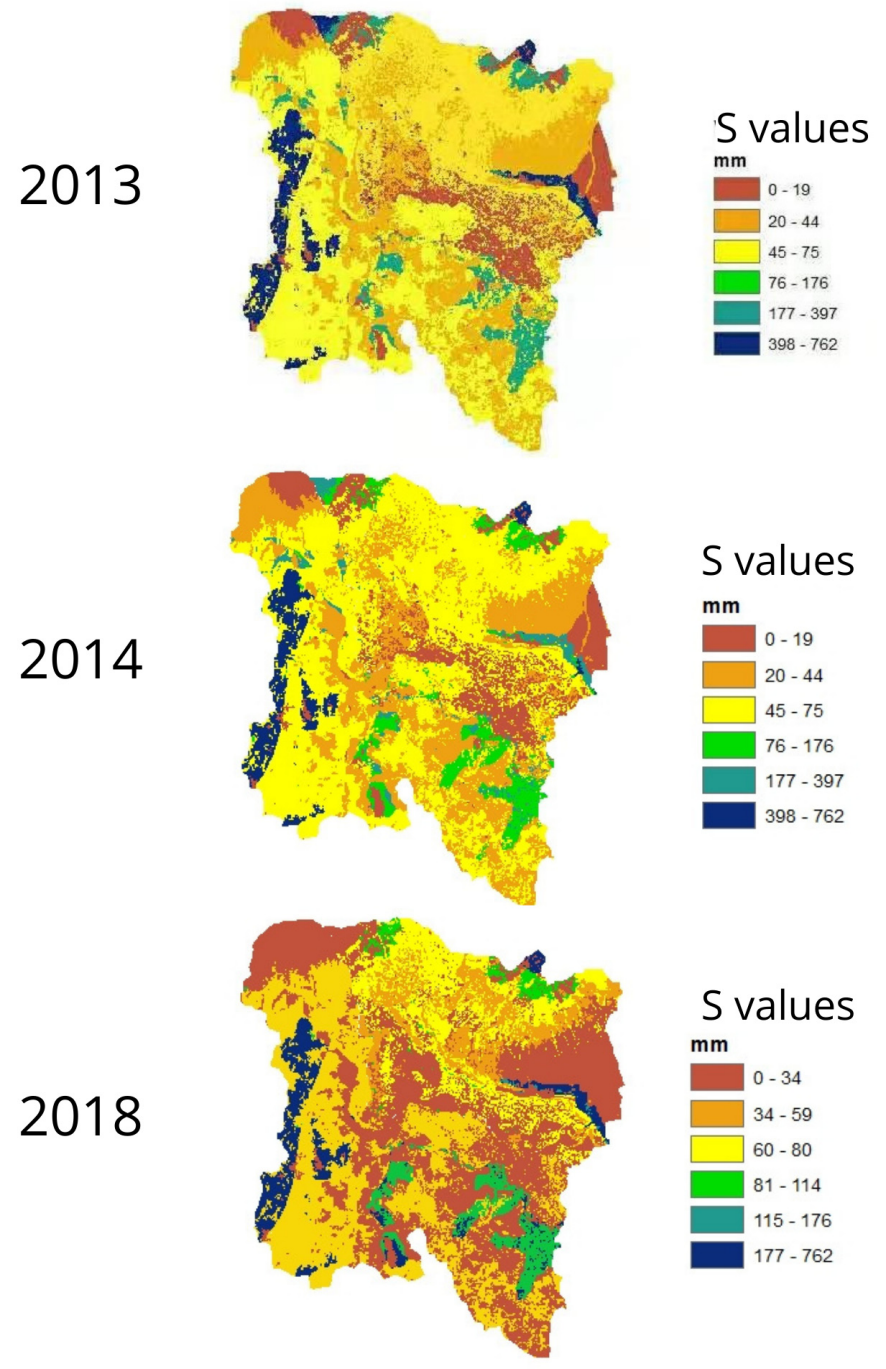
Maps of maximum retention potential of the Chibunga and Guano rivers micro-basins.

In Figure 2, the “S” values ( Lian *et al*., 2020) are shown for the Chibunga River micro-basin for years 2013 and 2014. One can observe a low CN value (orange color) in the range of 1–52 mm of water retained in the soil. Highest values of the water retention potential in the study area are highlighted in yellow; they correspond to moorlands and coniferous forests. In the micro-basin of the Guano River, we uncovered water retention potential values of 0 mm (red color). Such areas are characterized by the presence of urbanization, bodies of water, and snow. On the opposite end, highest values of water retention correspond to areas with eucalyptus forests (blue color), which are located in the middle part of the micro-basin. One should emphasize that the vegetation cover in this area has not changed over the three years when the multitemporal study was conducted.

### Precipitation-runoff ratio

**Figure 3.**
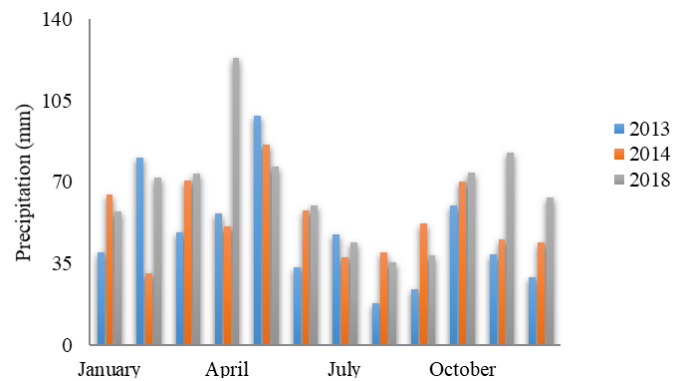
Average monthly precipitation.

In Figure 3, the variation of precipitation is shown over the 3 years of our multitemporal study. Our findings agree with a prior study of Chidichimo and co-workers, according to which highest precipitation occurs over the months of March, April, and May, while minimum precipitation values are recorded in January, November, and December ( Chidichimo *et al*., 2018).

The values of precipitation-runoff in the micro-basins were obtained via the curve number method (CN). Values of maximum retention potential were found to the vegetation cover for years 2013, 2014, and 2018.

**Figure 4.**
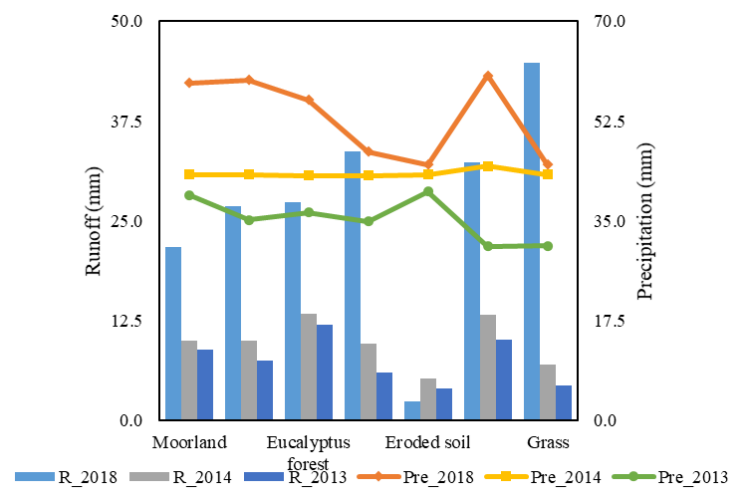
Precipitation-runoff relationship of the Chibunga river micro-basin.

In Figure 4, the precipitation-runoff relationship is much higher for an urban area in 2018 with a similar value of 44.6 mm, and a CN of 100, due to its retention capacity which is 0 . On the other hand, we obtained for vegetable crops and eucalyptus forests a similar value of 13 mm of runoff in 2014 and the retention capacity was of 60 and 68 mm of water in the soil, respectively. Therefore, it was determined that the grass category in 2013 had the lowest runoff at 4.4 mm and a CN of 79, demonstrating the saturation of porous medium in silty clay soil.

**Figure 5.**
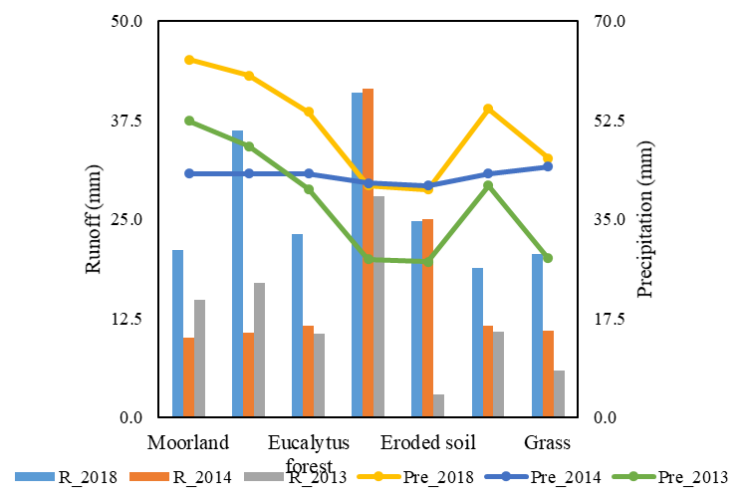
Precipitation-runoff relationship for the year 2014, 2013 and 2018 of the Guano river micro-basin.

Figure 5 reveals that in 2013, vegetable crops have a runoff of 12.7 mm and an average annual rainfall of 32.5 mm, while in 2014 and 2018, the urban area, wetlands, and eroded soil was in the range of 27–44 mm of runoff, showing retention of water of 0 mm in the soil. In addition, the greatest values of precipitation were found in 2018 for moorland category with 59.2 mm and runoff of 21.7 mm in the precipitation-runoff conversion, while the retention value is 72 mm of water, indicating the saturation of the porous medium in the soil.

## Conclusion

The maximum retention potential is directly affected by the CN values that are recorded according to the conditions and use of the vegetation cover on the land. The categories snow, and water, wetlands, and urban area showed null retention of water in the soil because their transformation is directly from precipitation to runoff. This trend was opposite for the other categories, with the highest values in the maximum retention potential, while their values of runoff decreased.

To better understand the water storage capacity, we had to adequately interpret the values of field capacity. This was done by relating the water storage capacity to permeability, porosity, and the amount of organic matter. Obtained values allowed the formulation of adequate recovery or conservation measures to achieve better retention of water in the soil.

## Data availability

### Underlying data

Figshare: GPS Coordinates of Sampling Points in Guano and Chibunga basins, https://doi.org/10.6084/m9.figshare.13874696.v1 ( Mendoza *et al*., 2021).

Figshare: Raw data Chibunga, https://doi.org/10.6084/m9.figshare.13875050.v1 ( Melendez, 2021).

Figshare: Supporting data: Guano (Recuperado).xlsx, https://doi.org/10.6084/m9.figshare.13299686.v1 ( Dama Research Center Limited, 2020).

Data are available under the terms of the Creative Commons Attribution 4.0 International license (CC-BY 4.0).
