# Supplementary material for: Determination of field capacity in the Chibunga and Guano rivers micro-basins
Source: F1000Res. 2021 Mar 3;10:172. [Version 1] doi: 10.12688/f1000research.28143.1 (PMC8630559; doi:10.12688/f1000research.28143.1)
Supplement: Peer review history

# Peer review: Reviewer response for version 1

**DOI:** 10.5256/f1000research.31130.r92491

**Published:** 2021-11-26

**Authors:** Dwita Sutjiningsih

**Affiliations:** 1 Department of Civil Engineering, Universitas Indonesia, Depok, Indonesia

The title should be added with the name of the country.

 In general, the work is clearly and accurately presented and cites the current literatures as well, except the presentation of tables and figures. The table should be accompanied by the unit dimension of the variables involved, for example in Table 1, precipitation is in mm. Figure 1 up to 3 are clear, but Figure 4-5 are a bit difficult to interpret, maybe it will be clearer if for each type of land cover, a relationship is made between precipitation and runoff.

 The study design is appropriate, and the work is also technically sound. Sufficient details of methods and analysis are provided to allow replication by others.

 Although it is presented in Spanish, the source data underlying the results are available and accessible to ensure full reproducibility.

 The conclusions drawn however are very general, so without conducting any research, one can make such a statement. Conclusions should be made more specific. Also should be synchronized with the conclusion section in the abstract.

**Note: **Comment from Reviewer 1 that is not yet responded: Which one is used? A total of 23 points corresponding to the Chibunga river micro-basin and 21 points for the Guano river micro-basin (in “Analysis in the Laboratory”) **OR** The 26 sampling points were in the Chibunga river micro-basin and 23 points in the Guano river micro-basin (in “Results and Discussion”).

Is the work clearly and accurately presented and does it cite the current literature?

Partly

If applicable, is the statistical analysis and its interpretation appropriate?

Yes

Are all the source data underlying the results available to ensure full reproducibility?

Yes

Is the study design appropriate and is the work technically sound?

Yes

Are the conclusions drawn adequately supported by the results?

Partly

Are sufficient details of methods and analysis provided to allow replication by others?

Yes

Reviewer Expertise:

NA

I confirm that I have read this submission and believe that I have an appropriate level of expertise to confirm that it is of an acceptable scientific standard.

# Peer review: Reviewer response for version 1

**DOI:** 10.5256/f1000research.31130.r80799

**Published:** 2021-03-23

**Authors:** Holger Benavides-Muñoz

**Affiliations:** 1 Universidad Técnica Particular de Loja (UTPL), Loja, Ecuador

**General comments:**

 The article is within the scope of the F1000 Research Open for Science.

 The abstract clearly explains the goal and the methodology as well as the flow of the paper. The method is well defined and well-illustrated as it is.

 This reviewer believes that the authors are using pertinent equations with relevant variables which are clear to understand; besides, the authors detailed the laboratory procedures used in analyzing the associated analytical data.

**Specific comments.-**

 On page 6, “The 26 sampling points were in the Chibunga river micro-basin and 23 points in the Guano river micro-basin.” should be replaced by “The 23 sampling points were in the Chibunga river micro-basin and 21 points in the Guano river micro-basin.” *Please, the authors should verify these numbers.*

Is the work clearly and accurately presented and does it cite the current literature?

Yes

If applicable, is the statistical analysis and its interpretation appropriate?

Yes

Are all the source data underlying the results available to ensure full reproducibility?

Yes

Is the study design appropriate and is the work technically sound?

Yes

Are the conclusions drawn adequately supported by the results?

Yes

Are sufficient details of methods and analysis provided to allow replication by others?

Yes

Reviewer Expertise:

Hydraulic and environmental engineering

I confirm that I have read this submission and believe that I have an appropriate level of expertise to confirm that it is of an acceptable scientific standard.
